# Extracorporeal Photopheresis with 5-Aminolevulinic Acid in Crohn’s Disease—A First-in-Human Phase I/II Study

**DOI:** 10.3390/jcm13206198

**Published:** 2024-10-17

**Authors:** Kristian Espeland, Eidi Christensen, Astrid Aandahl, Andreas Ulvær, Trond Warloe, Andrius Kleinauskas, Sagar Darvekar, Petras Juzenas, Vlada Vasovic, Qian Peng, Jørgen Jahnsen

**Affiliations:** 1 Department of Gastroenterology, Akershus University Hospital, N-1478 Lorenskog, Norway; jorgen.jahnsen@medisin.uio.no; 2 Department of Pathology, Norwegian Radium Hospital, Oslo University Hospital, N-0310 Oslo, Norway; eidi.christensen@ntnu.no (E.C.); trond.warloe@gmail.com (T.W.); andrius.kleinauskas@rr-research.no (A.K.); sagar.darvekar@rr-research.no (S.D.); petras.juzenas@rr-research.no (P.J.); vlada.vasovic@rr-research.no (V.V.); qian.peng@rr-research.no (Q.P.); 3 Institute of Clinical of Medicine, University of Oslo, N-0372 Oslo, Norway; 4 Department of Clinical and Molecular Medicine, Norwegian University of Science and Technology, N-7030 Trondheim, Norway; 5 Department of Dermatology, St. Olavs Hospital, Trondheim University Hospital, N-7030 Trondheim, Norway; 6 Department of Immunology and Transfusion Medicine, Akershus University Hospital, N-1478 Lorenskog, Norway; astrid.aandahl@ahus.no (A.A.); andreas.ulvaer@ahus.no (A.U.); 7 Department of Optical Science and Engineering, School of Information Science and Technology, Fudan University, Shanghai 200433, China

**Keywords:** Crohn’s disease, extracorporeal photopheresis, ECP, 8-MOP, UV-A, blue light, fecal calprotectin

## Abstract

**Background/Objectives:** With the increasing prevalence of Crohn’s disease (CD), treatment options for patients who fail conventional and advanced therapy are highly needed. Therefore, we explored the safety and efficacy of extracorporeal photopheresis (ECP) using 5-aminolevulinic acid (ALA) and blue light (405 nm). **Methods:** Patients with active CD who failed or were intolerant to biological therapy were eligible. Mononuclear cells (90 mL) were collected from each patient using a Spectra Optia^®^ apheresis system and diluted with 100 mL of 0.9% sodium chloride in a collection bag. The cells were incubated with ALA at a concentration of 3 millimolar (mM) for 60 min ex vivo and illumination with an LED blue light (405 nm) source (BLUE-PIT^®^) before reinfusion to the patient. Recording of vital signs and adverse events were regularly performed. At week 13, we assessed the patients with colonoscopy, the Harvey Bradshaw Index (HBI), the Inflammatory Bowel disease Health Related Quality of Life Questionnaire, and the measurement of serum C-reactive protein and fecal calprotectin (FC) levels. Biopsies of the intestines were taken for immunohistochemistry. **Results:** Seven patients were included. Four patients completed the treatments, with a total of 24 treatments. Three of the four patients achieved a favorable response, including a lower HBI, lower FC levels, and/or endoscopic improvement. No significant adverse events were observed. The remaining three patients received only one, three, or five treatments due to technical difficulties, medical reasons, or the withdrawal of informed consent. **Conclusions:** ALA-based ECP appears safe and seems to give some clinical improvement for the patients with active CD who failed to respond to conventional and advanced therapies.

## 1. Introduction

Crohn’s disease (CD) is a chronic, immune-mediated, inflammatory bowel disorder (IBD). The incidence is increasing globally, with reports of 10.6–29.3 per 100,000 people in the western world [1,2] and 0.09–3.91 per 100,000 people in low- and middle-income countries [3]. Young adults are primarily affected, and the disease has a large impact on morbidity and quality of life [1]. Its pathogenesis is complex and involves immune dysfunction, genes, the microbiome, and environmental factors [4,5,6]. In addition, T-cells are involved in this disease [7,8]. Current treatments for CD aim to inhibit or modulate the immune response in the gut by using drugs that affect different targets in the immune system [9].

In recent years, several biological drugs (tumor necrosis factor-a (TNF) inhibitors, a4b7 integrin, Interleukin (IL) 12/23, and IL23 inhibitors) and drugs targeting small molecules (Janus kinase (JAK) inhibitors and sphingosine-1-phosphate receptor (S1PR) modulators) have been introduced in clinical practice, and an increasing number of patients with CD have achieved satisfactory disease control. However, many patients do not improve and/or tolerate available treatments. Therefore, there is a need for alternative therapeutic methods.

Extracorporeal photopheresis (ECP) with the photosensitizer 8-methoxypsoralen (8-MOP) is a well-known and well-tolerated treatment for several T-cell-mediated diseases, including chronic graft versus host disease (cGvHD) and cutaneous T-cell lymphoma (CTCL) [10]. ECP combines the collection of leukocytes with the addition of 8-MOP as a photosensitizing agent before illumination with ultraviolet light A (UV-A). Since 8-MOP binds to the DNA of activated (diseased) and resting (normal) T-cells, traditional ECP damages both of these types of cells [11]. As CD is mediated by T-cells [7,8,12], the use of ECP for the treatment of CD has been explored in several small, uncontrolled studies, with some promising results in steroid-dependent and medically refractory cases [13,14,15].

The use of 5-aminolevulinic acid (ALA), a photosensitizer precursor, may be an alternative to 8-MOP with ECP, since it selectively targets cells, such as activated T-cells or cancer cells, and may improve the treatment effect [16]. ALA leads to the accumulation of the photoactive porphyrin protoporphyrin-IX (PpIX), and this porphyrin accumulation occurs to a lesser extent in resting or normal cells, which are unharmed after light exposure [17,18,19]. The absorption peak of PpIX is in the visible blue light range (400–420 nm) [20], which is the rationale for the use of blue light instead of UV-A light. Photodynamic diagnosis using ALA is a well-known option for the diagnosis of brain tumors and bladder cancer [21,22,23,24]. Further, photodynamic therapy with topical ALA application is a widely used topical treatment method for nonmelanoma skin cancer [25,26]. We have previously explored the use of ECP combined with ALA and UV-A light for patients with cGvHD or CTCL, and the results indicated that the ALA-ECP treatment was safe [27,28].

Therefore, the present study investigated the safety and efficacy of ECP combined with ALA and blue light (405 nm) in patients with active CD who have failed or were intolerant to advanced therapy. In addition, we wanted to perform immunohistochemistry on biopsies from the ileum and the sick and presumed healthy colons in the patients before and after the treatment.

## 2. Materials and Methods

### 2.1. Study Design and Approvals

This study is the first-in-human open-label phase I/II study to explore the safety and efficacy of ALA-ECP in patients with active CD. The study was approved by the ethics committee (REK—reference number 15685), the Norwegian Medicines Agency (NOMA—EudraCT 2018-002422-23, NOMA reference number 18/10491), and the Data Protection Official (DPO, reference number 78_2019) at the Akershus University Hospital. It was registered on ClinicalTrials.gov (NCT04164849).

### 2.2. Patient Population

Patients with active CD and failure or intolerance to advanced therapy at the Akershus University Hospital from November 2019 to March 2023 were considered for the study inclusion. Active CD was defined as fecal calprotectin (FC) > 250 mg/kg and/or serum C-reactive protein (CRP) > 5 mg/L, in addition to a Harvey Bradshaw Index [29] (HBI) > 5 points and endoscopy with a simple endoscopic score for Crohn’s Disease [30] (SES-CD) ≥ 6 or ≥4 points if only isolated ileitis was present. Informed consent for study participation was obtained before inclusion. The patients were assessed using colonoscopy, the HBI, clinical workup, blood samples, and the Inflammatory Bowel Disease Quality of Life Questionnaire (IBDQ) [31,32] at screening. The washout period for biological drugs was 2 months, and steroids were tapered for at least 2 weeks before entering the study. Immunosuppressant drugs (methotrexate, mercaptopurine, and azathioprine) could be continued if at a stable dose for at least 8 weeks. Women of childbearing potential were obliged to use highly effective contraceptives throughout the study, and to perform a pregnancy test at screening and before all treatment visits.

The exclusion criteria for the study were as follows: patients with photosensitive comorbidities, porphyria, or known hypersensitivity to 5-aminolevulinic acid or porphyrins; individuals with aphakia; pregnant or breastfeeding women (a negative urine pregnancy test was required for women of child-bearing potential at the screening visit and before each treatment); patients with ongoing cardiac or pulmonary diseases, abnormal ASAT, ALAT, bilirubin, or INR values (≥3× upper limit of normal), or clinically significant ECG findings; individuals with polyneuropathy; uncontrolled infection or fever; a history of heparin-induced thrombocytopenia, an absolute neutrophil count <1 × 10^9^/L or a platelet count <20 × 10^9^/L; a body weight below 40 kg; subjects deemed unlikely to comply with study procedures by the investigator; those with other gastrointestinal diseases that could potentially influence the study outcomes.

A history of any clinically significant disease or disorder which, in the opinion of the investigator, may either put the patient at risk because of participation in the study or influence the result or the patient’s ability to participate in the study was also considered.

### 2.3. Treatment Drug

ALA (Gliolan^®^, Photonamic GmbH & Co, KG, Pinneberg, Germany) was obtained in accordance with Good Clinical Practice (GCP). ALA was mixed on a laminar flow bench by adding 50 mL of sterile 0.9% sodium chloride (Fresenius Kabi, Hamburg, Germany) to a vial, followed by gentle shaking. After mixing, the vial was stored at 4 °C prior to use before 6 p.m. the next day.

### 2.4. Treatment Procedures

**Figure 1:**
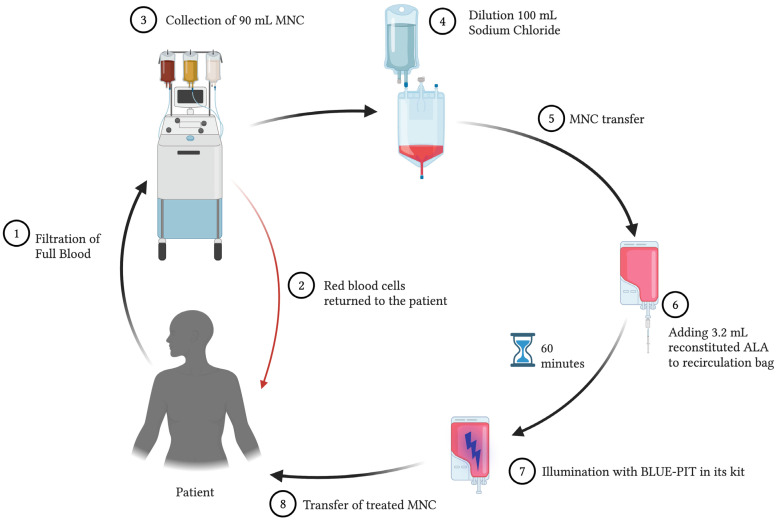
Overview of the treatment setup. A volume of 90 mL of MNCs was collected in the Spectra Optia^®^ with the Continuous Mononuclear Cell Collection protocol, diluted, and transferred to the BLUE-PIT kit. ALA was added through a sterile filter (0.2 μm) to the BLUE-PIT device. After ALA incubation, followed by illumination, the cells were returned to the patient as a blood transfusion through a standard 200-μm filter. Created in Biorender. Espeland, K. (2024). BioRender.com/l41k528 (accessed on 10 October 2024).

Treatment was performed using a Spectra Optia^®^ apheresis system (Terumo BCT, Tokyo, Japan) with a continuous mononuclear cell collection protocol. A volume of 90 mL of mononuclear cells (MNCs) was collected and diluted with 100 mL of 0.9% sodium chloride. A total of 190 mL of the diluted MNC solution was transferred to a recirculation bag connected to the BLUE-PIT light kit (PIT Medical Systems, Cadolzburg, Germany). The recirculation bag and lines were shaded from room light. Upon transfer, and throughout the rest of the procedure, the patient remained connected to the Spectra Optia^®^, and no lines were cut to maintain a closed-circuit system. A volume of 3.2 mL of the reconstituted ALA was added to the MNC solution in the recirculation bag through a sterile filter. Because the light dose in the BLUE-PIT light device is automatically determined by the hematocrit concentration in the MNC solution, a standardized high hematocrit of 1.5% was set in the device to ensure sufficient light exposure in all of the treatments. The BLUE-PIT device slowly circulated the MNC solution that was mixed with ALA for 1-h to produce intracellular PpIX before the BLUE-PIT light device automatically illuminated the MNCs with blue light (405 nm). The ALA dose and exposure time were set according to our previous research [16,18]. After the light exposure, the treated MNCs were transfused back to the patient through a standard transfusion filter (CODAN Medizinische Geräte GmbH & Co., Lensahn, Germany). The transfusion bag remained covered from room light until all of the MNCs were transfused back to the patient. The entire procedure took place on one single day. Figure 1 shows a schematic overview of the treatment procedure.

The treatment was performed once every 2 weeks for 10 weeks in accordance with the guidelines for clinical studies on CD [33]. Thirteen weeks following the initial treatment, the patients underwent evaluation, with the screening visit serving as the baseline reference.

### 2.5. Safety Assessments

Safety was monitored using the frequency and severity of adverse events (AEs), vital signs (blood pressure, pulse, and body temperature before treatment, after apheresis but before transfusion, and after transfusion), physical examination, and hematological and clinical laboratory measurements. Urine was examined using a dipstick for erythrocytes, glucose, ketone, leukocytes, nitrite, pH, and protein. A 12-lead electrocardiogram (ECG) was recorded at screening and week 13.

Additionally, the patients were monitored with a 3-lead ECG during all treatments.

### 2.6. Treatment Assessments

Our primary endpoint was the clinical response (HBI reduction > 3 points) at week 13.

Our secondary endpoints were clinical remission (HBI < 5 points) and any reduction in the FC and CRP from the baseline, in addition to the IBDQ, at week 13. We also performed ileocolonoscopy with the SES-CD at screening and 13 weeks after the treatment to assess the endoscopic response. The endoscopic effect was defined as a ≥50% reduction from the baseline. The following biopsies were also obtained: 4 from the ileum and 4 from endoscopically inflamed and endoscopically normal mucosa in the colon. The biopsies were immediately frozen and stored at −150 °C for the immunohistochemistry analysis of CD4 for T helper cells and FOXP3 for regulatory T-cells (Tregs).

### 2.7. Immunohistochemistry

The frozen tissue biopsies were thawed and then subjected to formalin fixation and paraffin embedding. Three-micron-thick paraffin-embedded tissue sections were cut and mounted on positively charged glass microscope slides. Deparaffinization was performed using the PT-link and EnVision^TM^ FLEX target retrieval solution (with a high pH) (Dako, Glostrup, Denmark). To block endogenous peroxidase activity, the sections were treated for 5 min with EnVision Peroxidase-Blocking reagent (Dako) prior to a 30-min incubation with mouse anti-CD4 or anti-FOXP3 primary antibody (Dako IR649 and Abcam ab20034). The sections were treated for 15 min with EnVision FLEX+ mouse (SM804) antibody, and then 30 min with horseradish peroxidase (HRP)-labeled anti-mouse secondary antibody, followed by 10 min with 3′3 diaminobenzidine tetrahydrochloride (DAB) before staining with Hagen’s hematoxylin. The tissue sections were also stained with routine hematoxylin and eosin (H&E). The sections were dehydrated and mounted in Pertex from Histolab.

### 2.8. Statistics

The data are presented as the mean (minimum–maximum). Vital signs (heart rate, systolic and diastolic blood pressure, and temperature) are presented in violin plots before apheresis, after apheresis, and after transfusion of the illuminated MNCs to the patient. The violin plots were created using Stata/SE 18.0 for Mac (StataCorp, Lakeway, TX, USA).

## 3. Results

### 3.1. Patients and Treatment

Seven patients were included in the study, of which four completed the 10-week treatment.

The demographics, disease extent according to the Montreal Classification [34], and information about the biologics used for these four patients at screening are shown in Table 1.

### 3.2. Treatment Data

The four patients received 6 treatments each, giving a total of 24 treatments. The mean (minimum–maximum) treatment duration from the initiation of the MNC collection to the reinfusion of treated MNCs was 232.5 min (200–295). A total of 90 mL of MNCs were collected, and 100 mL of 0.9% sodium chloride was added to the MNC solution. The light dose was standardized to 13.7 Joules for 23 of the 24 treatments by setting the same 1.5% hematocrit to the BLUE-PIT device. In the first treatment of the first patient, the light dose was 6 Joules due to the setting of a measured hematocrit of only 0.5%.

The differential counts and hematocrit of the diluted MNCs were measured before the addition of ALA in all of the treatments, and contained high levels of lymphocytes and monocytes and low levels of red blood cells (Table 2).

Three patients did not complete the full course of treatment due to technical issues, medical reasons, or the withdrawal of informed consent. These patients received one, three, and five treatments, respectively.

### 3.3. Safety

The analysis of the blood samples at all time points showed small variations within the reference values. There were no significant changes over time in the four patients. The values of the liver tests and creatinine are presented in Table 3.

**Figure 2:**
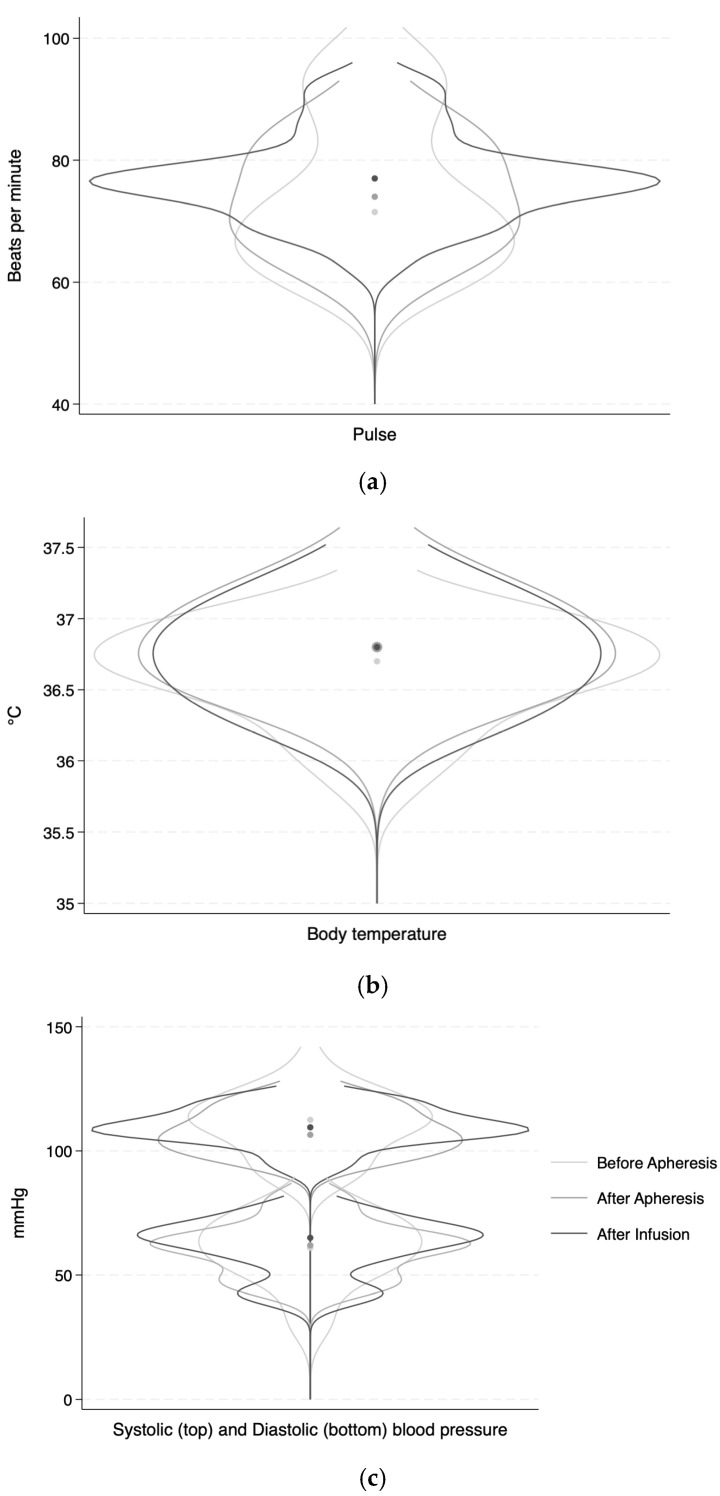
Vital signs. (**a**) Pulse (rate per minute), (**b**) body temperature (°C), and (**c**) systolic and diastolic pressure (mmHg) before apheresis, after apheresis but before transfusion, and immediately after transfusion of the product to the patient. The central dots are the average.

The heart rate, blood pressure, and body temperature varied slightly within each patient during the treatment. Figure 2 shows the variations presented as overlaying violin plots.

No serious adverse events were recorded for the patients during the study period. The total number of AEs was 23, all of which were considered low grade according to the Common Terminology Criteria for AEs (CTCAE version 4.03).

Table 4 summarizes all of the AEs.

Adverse events occurring in the different patients (numbers 1–4) throughout the study, and whether they were deemed to be related to the ALA. The grade according to the CTCAE (1–5).

There were no serious adverse events recorded in the patients not completing the treatment.

### 3.4. Efficacy

Of the four patients who completed the treatment, three demonstrated some degree of clinical improvement; however, none achieved full clinical remission. A reduction in SES-CD ≥ 50% and a lower FC from the baseline was demonstrated in two of three patients. In patient 1, no clinical or endoscopic changes were observed after six treatments. The changes in the IBDQ score were small and not significant, but correlated with the treatment effects (Table 5).

### 3.5. Immunohistochemistry

**Figure 3:**
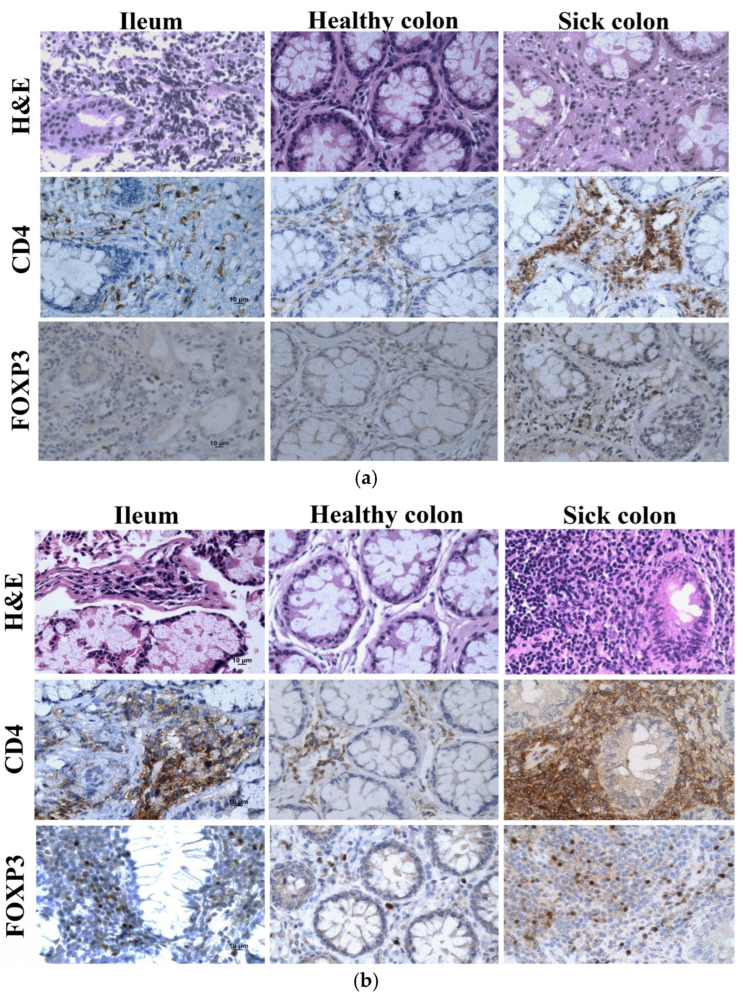
Histopathology (H&E) and immunohistochemistry (CD4 and FOXP3) images of the ileum, healthy colon, and sick colon taken from patient 2 with CD before (**a**) and at week 13 (**b**) after the ALA-ECP. The scale bars are 10 µm for all images.

The tissue sections of the ileum and healthy and sick colon exhibited lymphoid infiltration, with lymph follicles in the lamina propria that likely extended into the submucosa through the incomplete muscularis, but there was less lymphocyte infiltration in the healthy colon than the ileum and sick colon (Figure 3a). Immunohistochemistry of the surface marker CD4 for helper T-cells and the transcription factor forkhead box protein 3 (FOXP3) for regulatory T-cells (Tregs) confirmed the infiltration of CD4^+^ T-cells and Tregs largely in the lymph follicles. Because there was more lymphoid infiltration in the ileum and sick colon, there were more CD4^+^ cells and Tregs in these samples. The samples from the ileum and sick colon taken 13 weeks after treatment appeared to have more CD4^+^ T-cells and Tregs than the samples taken before the treatment (Figure 3b). However, there was a large variation in the amounts and distribution patterns of these cells in different biopsies of the same area and various areas of the same and different patients.

## 4. Discussion

In this first-in-human study, clinical and endoscopic efficacy was demonstrated in patients with active CD treated with the ALA/blue-light-based ECP. All of the participants had previously failed biological therapies due to intolerance or perceived contraindication. No safety or tolerability concerns were observed.

Overall, the patients tolerated the treatment very well. However, two episodes of nausea, which is a common side effect of ALA, were recorded, and there were also a few episodes of headache. All of the reported side effects were self-limiting without medical intervention.

Although conventional 8-MOP/UV-A-based ECP has been investigated in some small studies for the treatment of CD with effects for steroid-refractory cases and patients intolerant or unresponsive to other options [13,14,15,35], a disadvantage of the DNA binding of 8-MOP is the ability to kill both activated (diseased) and resting (normal) cells with no selectivity. Since our previous research has revealed greater selective killing effects on T-cells after treatment with ALA/blue light than after treatment with 8-MOP/UV-A [16,18], a two-step approach for ECP was used in this study, with the possibility of using the Spectra-Optia^®^ for MNC collection, followed by illumination with a separate 405-nm blue light source, which is suitable for the absorption peak of PpIX.

The disadvantages of using ALA/blue light was the treatment time of approximately 4 h, in addition to patient logistics, including transportation and the gaining of intravenous access. Regular ECP using 8-MOP with UV-A is faster [36,37], partially due to the ALA incubation. However, the ALA/blue light ECP method could potentially be enhanced in the future by simplifying the procedure, for example, by dividing it into the following three distinct phases: collection, incubation, and transfusion. After the collection phase, the patient would be free during the incubation period, which could be extended. A longer incubation period may allow for the greater accumulation of PpIX [17,18], potentially leading to an improved photodynamic effect.

Overall, autoimmune diseases, including CD and GvHD, represent common immune-mediated diseases with diverse clinical presentations. These diseases share common underlying pathogenetic features but present their own clinical immune phenotypes. Tregs produce suppressive messengers, such as TGF-beta and IL-10, to inhibit the activation, proliferation, and cytokine production of other cells in the immune system. They regulate immune responses to self-antigens and foreign antigens and help prevent immune-mediated diseases. Therefore, Tregs are indispensable for the establishment and maintenance of immunological self-tolerance and homeostasis. FOXP3 is an essential molecular marker in the development and function of Tregs [38]. Because the reduced number and functional abnormalities of tolerogenic Tregs may cause immune-mediated diseases [39], it is tempting to investigate whether Tregs are involved in the treatment of CD with ALA-ECP. A decrease in Tregs may be associated with active IBD [40]. There were more CD4^+^ T-cells and Tregs in the ileum and sick colon at week 13 after ALA-ECP than before the treatment, which suggests that ALA-ECP enhances the infiltration of Tregs into diseased tissues and improves Treg infiltration. However, there is large variation in the amounts and distribution patterns of Tregs in different biopsies of the same area of the same patient and between different patients. Further studies with more CD patients are needed to confirm these findings.

One of the main limitations in our study is the small samples size, which limits the conclusions that can be drawn from this study. Further limitations are the lack of a control group and the lack of a blinded follow-up. In this study, we experienced a long inclusion period. This was due to the COVID-19 pandemic, which led to the postponement of the inclusion of patients and to the change in work tasks for the study team. Previously, no randomized clinical study on ECP in patients with CD has been performed, although ECP guidelines list it as an available option [10,41]. On the other hand, the European Crohn’s and Colitis Organization (ECCO) guidelines do not mention this treatment method as an option [9]. Even so, we argue that ECP could have a place in CD treatment. With the increasing prevalence of CD, more patients will fail or be intolerant to conventional and/or advanced therapy. A particularly important group of patients are the patients with a history of malignancies for whom treatment with immunosuppressive drugs is often perceived as contraindicated. Additionally, ECP has few side effects, including a lower risk of infection, which is a common adverse effect associated with biologics.

Furthermore, combination therapy for CD is a topic of significant international interest [1,42]. ALA/blue light ECP may be an option in induction therapy for hospitalized critically ill patients to help reboot the immune system, but this possible treatment option must be explored in clinical studies.

In conclusion, the results of this study suggest that six single-day ALA/blue-light-based ECP treatments over 10 weeks is both safe and well-tolerated, with the potential to provide clinical efficacy in patients with CD. These findings provide a basis for further investigation into the use of ALA-ECP in the treatment of CD and other T-cell-mediated disorders. A particular group of interest could be patients with co-existing malignancies, in whom advanced immunosuppressive therapies are not recommended due to the lack of knowledge regarding safety.

## Figures and Tables

**Table 1:** Patient population at screening.

Patient	Age	Sex	CD Diagnosis	Disease Extent	Biologics Previously Used
1	36	M	2002	Ileocolic	IFX + ADA + VDZ + UST
2	49	F	1994	Ileocolic	IFX + VDZ ^(i)^
3	54	F	1984	Ileocolic	IFX + ADA
4	62	F	2003	Ileal	IFX ^(ii)^

^(i)^ Perceived contraindication with other options due to previous breast cancer. ^(ii)^ Perceived contraindication with other options due to melanoma and lymphoma (both treated). IFX = infliximab; ADA = adalimumab; VDZ = vedolizumab; UST = ustekinumab.

**Table 2:** Differential counts of the diluted products.

Hematocrit (%)	0.31 (0.05–0.80)
Hemoglobin (g/dL)	0.17 (0.0–0.3)
White Blood Cells (10^9^/L)	23.94 (8.7–37.5)
Neutrophils (10^9^/L)	0.89 (0.00–7.9)
Lymphocytes (10^9^/L)	18.92 (5.15–33.19)
Monocytes (10^9^/L)	4.17(0.6–6.9)
Platelets (10^9^/L)	379 (60–997)

Differential counts of the diluted products presented as the mean (minimum–maximum) of all 24 treatments in four patients.

**Table 3:** Liver and kidney parameters.

	Reference Values	1		2		3		4	
		Sc.	Week 13	Sc.	Week 13	Sc.	Week 13	Sc.	Week 13
ALT	10–45 U/L	17	18 (15–20)	14	13 (11–15)	18	14 (11–18)	20	23 (21–25)
AST	15–35 U/L	19	20 (15–28)	22	21 (19–22)	21	22 (19–23)	24	25 (22–28)
ALP	35–105 U/L	73	78 (57–96)	50	45 (40–47)	43	46 (41–49)	76	81 (74–93)
GT	10–45 U/L	29	27 (17–38)	13	13 (10–17)	7	7 (6–8)	22	19 (16–22)
Albumin	36–48 G/L	43	42 (39–45)	40	41 (39–42)	42	41 (40–43)	48	46 (44–48)
INR	0.8–1.2	1.0	1 (0.9–1.1)	1.1	1.1 (1.1–1.2)	1.0	0.9 (0.9–1)	1.1	1.1 (1.1–1.1)
Creatinine	45–90 μmol/L	88	90 (77–103)	61	60 (55–62)	71	77 (74–82)	92	88 (84–95)

The results are presented with the screening values compared to the values before each treatment visit and the assessment at week 13. The week 0–13 data are presented as the mean (minimum–maximum). The bolded parameters (GT, creatinine) were slightly less (GT) and above (creatinine) the reference values and were assessed as not clinically significant. Sc. = screening; ALT = alanine aminotransferase; AST = aspartate aminotransferase; ALP = alkaline phosphatase; GT = glutamyl transferase; INR = international normalized ratio.

**Table 4:** Frequency and severity of adverse events.

Adverse Event	Patients	Number of Events (Grade)
Headache	1, 3	4 (1)
Nausea	2, 3	2 (1)
Fistulae	3	3 (1)
Neck/back pain/general pain/shoulder pain	1, 2, 3	5 (1)
Fever	2	1 (1)
COVID-19/upper airway infection	2, 3	4 (1)
Glucosuria	4	1 (1)
Urinary tract infection	2	1 (1)
Tachycardia	3	1 (1)
Heartburn	3	1 (1)

**Table 5:** Treatment effects.

	Visit	1	2	3	4
HBI	Screening	10	6	6	24
	Week 13	22	2	2	4
SES-CD	Screening	14	17	19	9
	Week 13	14	8	8	3 ^(iii)^
FC	Screening	949	500	2580	429
	Week 13	1331 ^(i)^	115	3700	208
IBDQ	Screening	135	163	175 ^(ii)^	184
	Week 13	102	188	199	199

Showing the treatment effects at screening and at week 13 in the four patients that completed the six treatments. The reference values for the HBI were as follows: remission < 5, mild disease 5–7, moderate disease 8–16, and severe disease > 16. FC: remission < 150. SES-CD: remission < 3, mild disease 3–6, moderate disease 7–15, and severe disease > 15. IBDQ: remission > 170 [26]. ^(i)^ Taken earlier due to the worsening of the disease (week 7); ^(ii)^ Question 27 is missing; ^(iii)^ The colonoscopy was postponed because the patient was not able to return. The FC at week 16, when the colonoscopy was performed, was 159.

## Data Availability

The original contributions presented in the study are included in the article, and further inquiries can be directed to the corresponding author.
